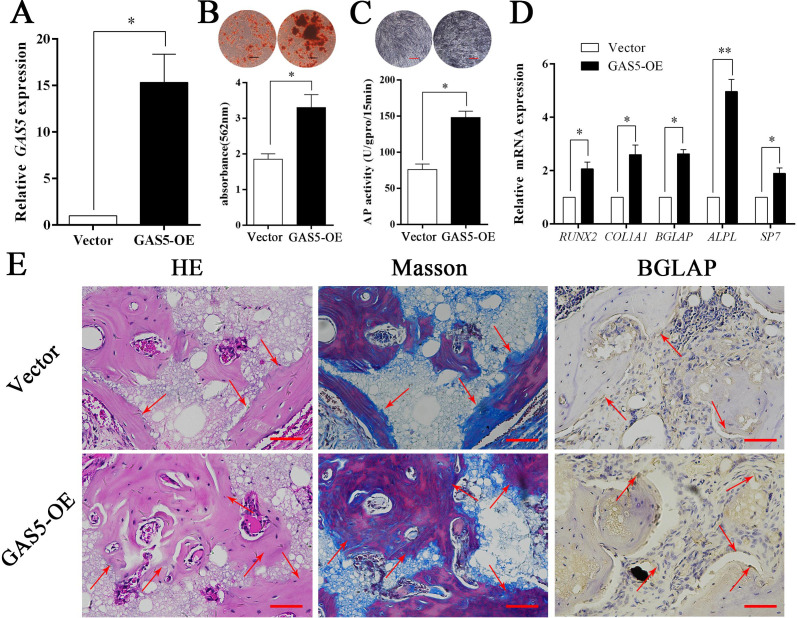# Correction: GAS5 protects against osteoporosis by targeting UPF1/SMAD7 axis in osteoblast differentiation

**DOI:** 10.7554/eLife.98211

**Published:** 2024-04-25

**Authors:** Ming Li, Zhongyu Xie, Jinteng Li, Jiajie Lin, Guan Zheng, Wenjie Liu, Su'an Tang, Shuizhong Cen, Guiwen Ye, Zhaofeng Li, Wenhui Yu, Peng Wang, Yanfeng Wu, Huiyong Shen

**Keywords:** Human, Mouse

Li M, Xie Z, Li J, Lin J, Zheng G, Liu W, Tang S, Cen S, Ye G, Li Z, Yu W, Wang P, Wu Y, Shen H. 2020. GAS5 protects against osteoporosis by targeting UPF1/SMAD7 axis in osteoblast differentiation. *eLife* **9**:e59079. doi: 10.7554/eLife.59079.

Published 2 October 2020

We recently discovered several errors in our published article. Figure 5M (middle panel) is a duplication of Figure 3C (right panel). This error is likely due to the similarity of the images for both panels, with the original raw images are stored in adjacent computer folders. Figure 5M (middle panel) now shows the correct ALP staining image. We also noticed errors in the annotation of the symbols beneath Figure 5L and Figure 5M. The ‘+’ symbol in row 4, column 2 with a ‘-’ symbol. Additionally, we removed redundant Figure 3-figure supplement 1, because it is identical to Figure 2-figure supplement 1. The text has been corrected accordingly.

We have also updated the published paper to now include the raw microscopy images from Figure 3 and Figure 5 as Figure 3—source data 1 and Figure 5—source data 1:

Figure 3—Source data 1. Original images of alizarin red staining, ALP staining, and in vivo HE staining and Masson staining in GAS5 overexpression group and control group.

Figure 5—Source data 1. Original images of alizarin red staining and ALP staining in experiments of ‘GAS5 interacts with UPF1 to accelerate SMAD7 decay’.

The article has been corrected accordingly. The updated figures are shown below.

The corrected Figure 5 is shown here:

**Figure 1:**
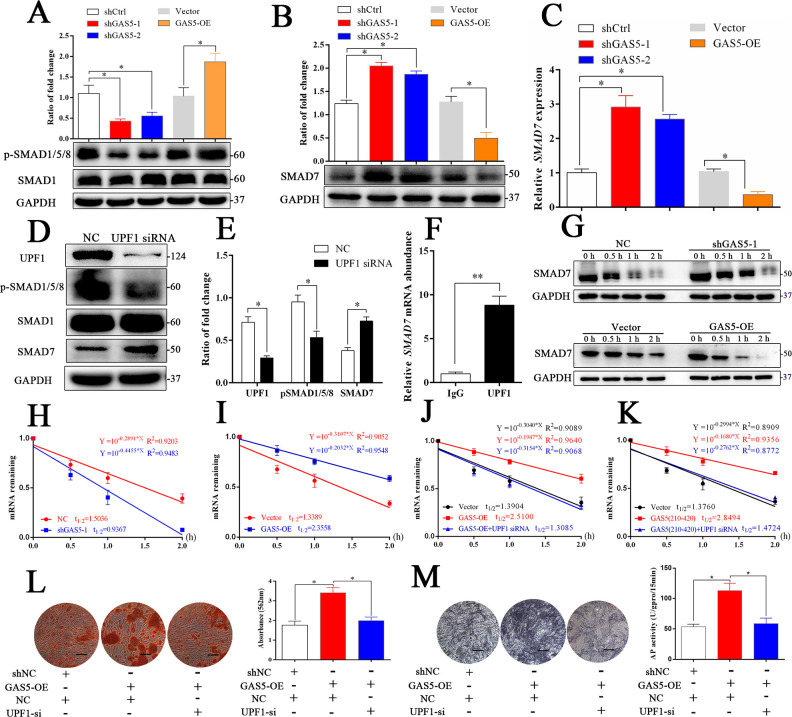


The originally published Figure 5 is shown here for reference:

**Figure 2:**
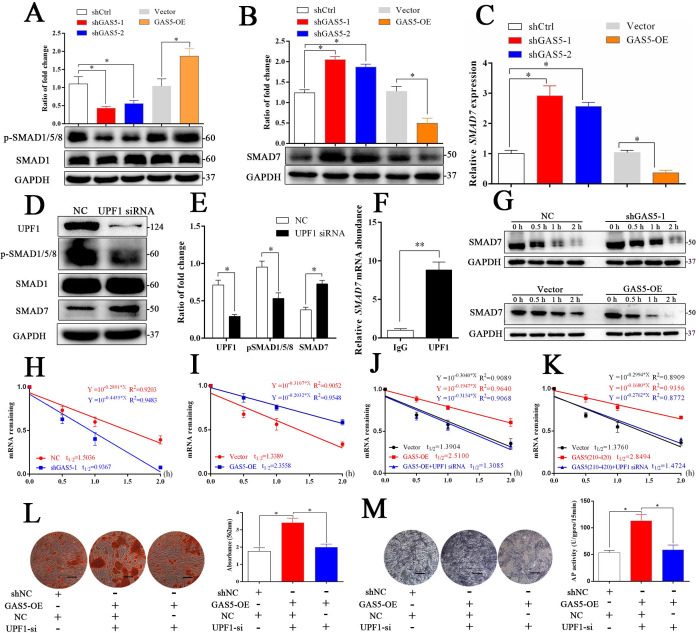


The originally published Figure 3 is shown here for reference:

**Figure 3:**