# Supplementary figures and images for: GAS5 protects against osteoporosis by targeting UPF1/SMAD7 axis in osteoblast differentiation
Source: eLife. 2020 Oct 2;9:e59079. doi: 10.7554/eLife.59079 (PMC7609060; doi:10.7554/eLife.59079)

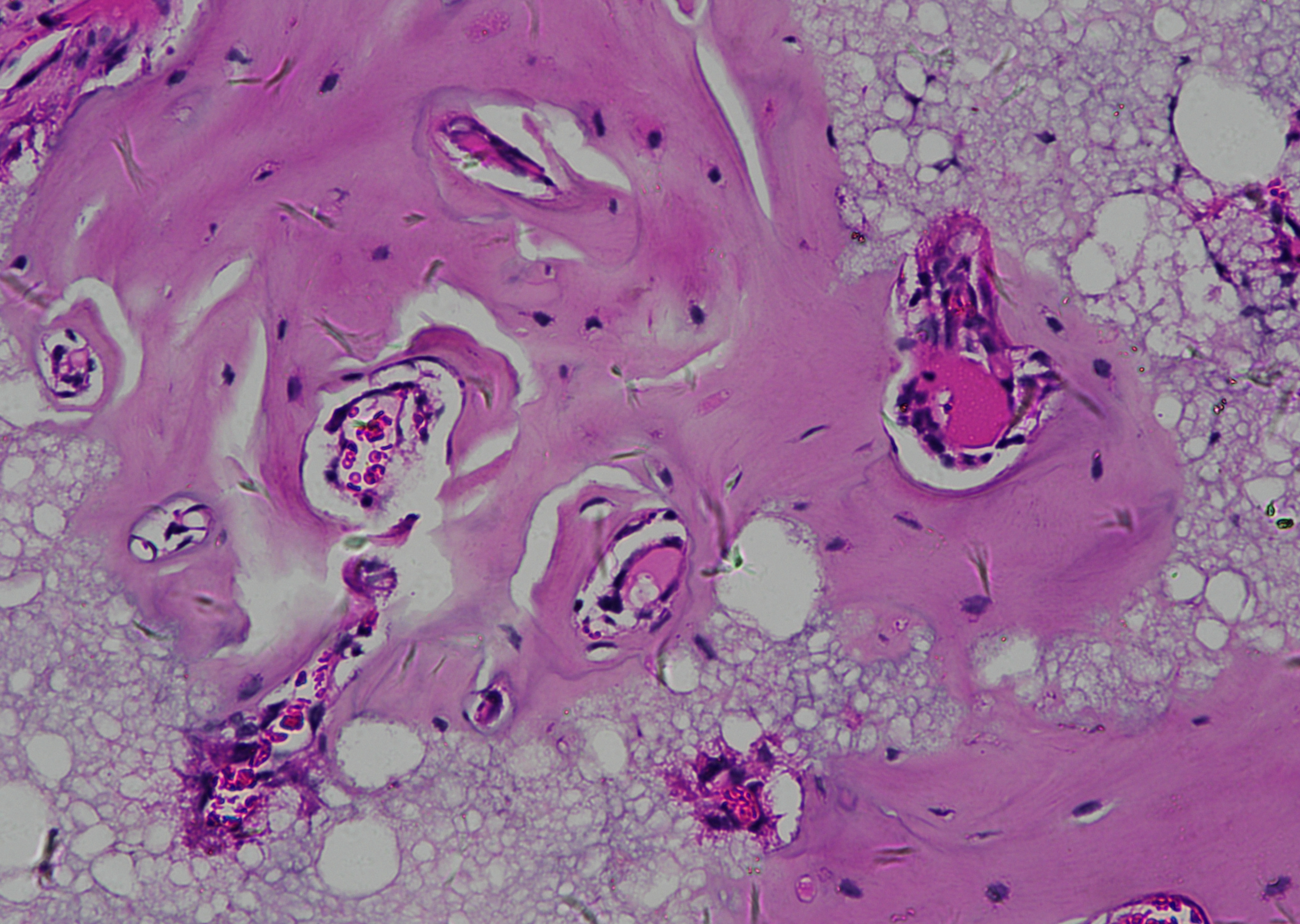

Supplement: Figure 3—source data 1. [file elife-59079-fig3-data1.zip › HE-GAS5-OE.tif]

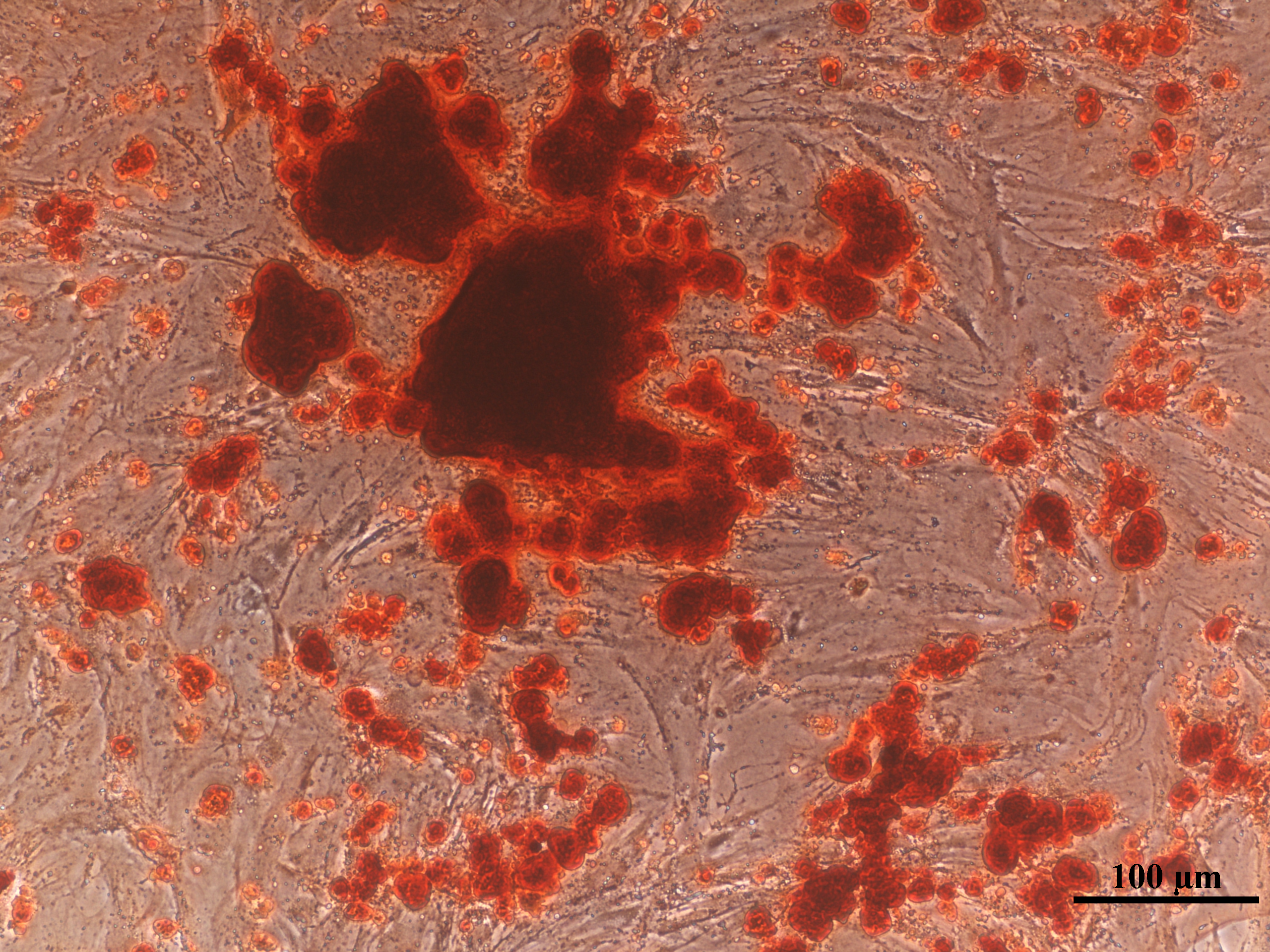

Supplement: Figure 3—source data 1. [file elife-59079-fig3-data1.zip › ARS-GAS5-OE.tif]

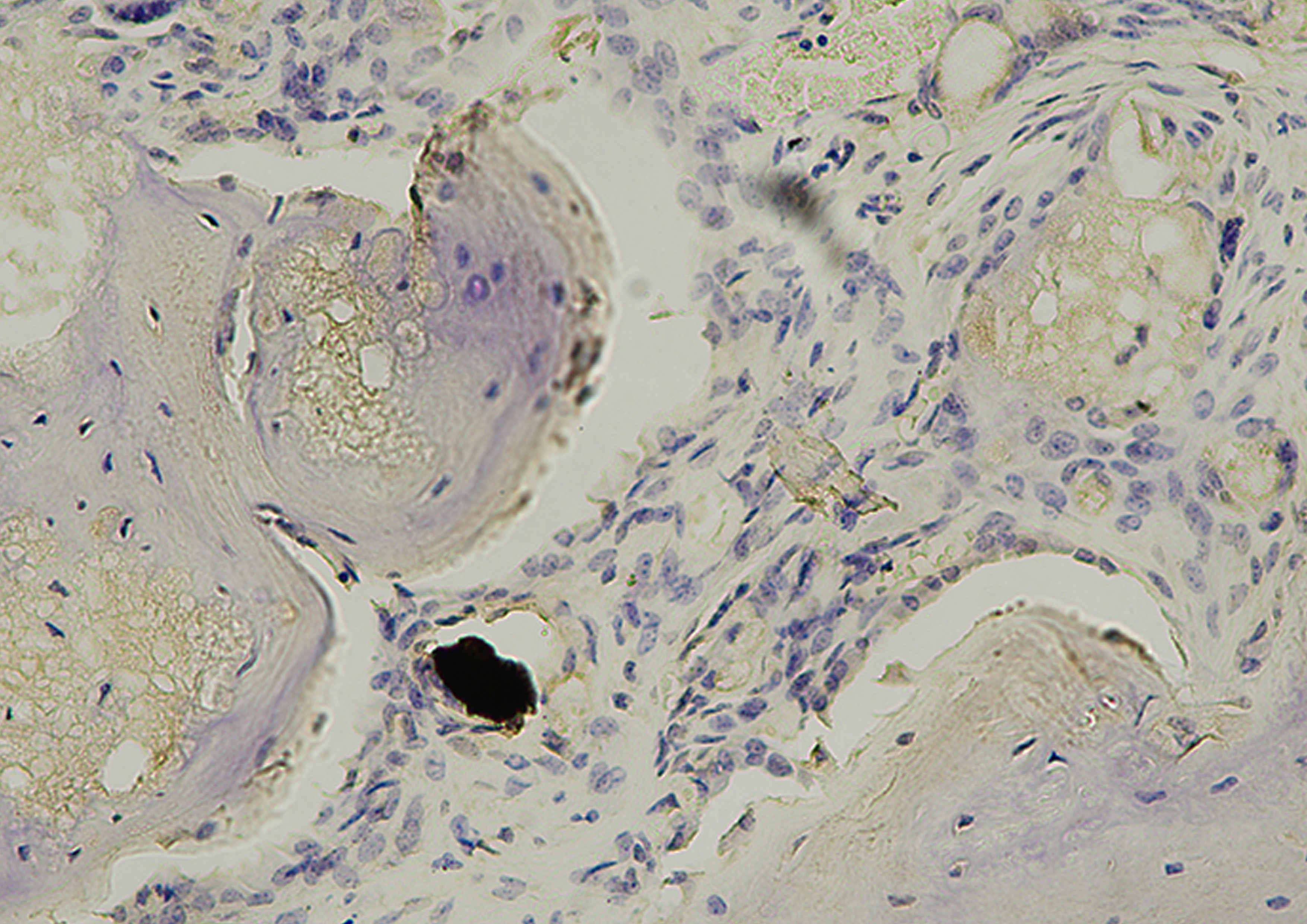

Supplement: Figure 3—source data 1. [file elife-59079-fig3-data1.zip › OCN-GAS5-OE.tif]

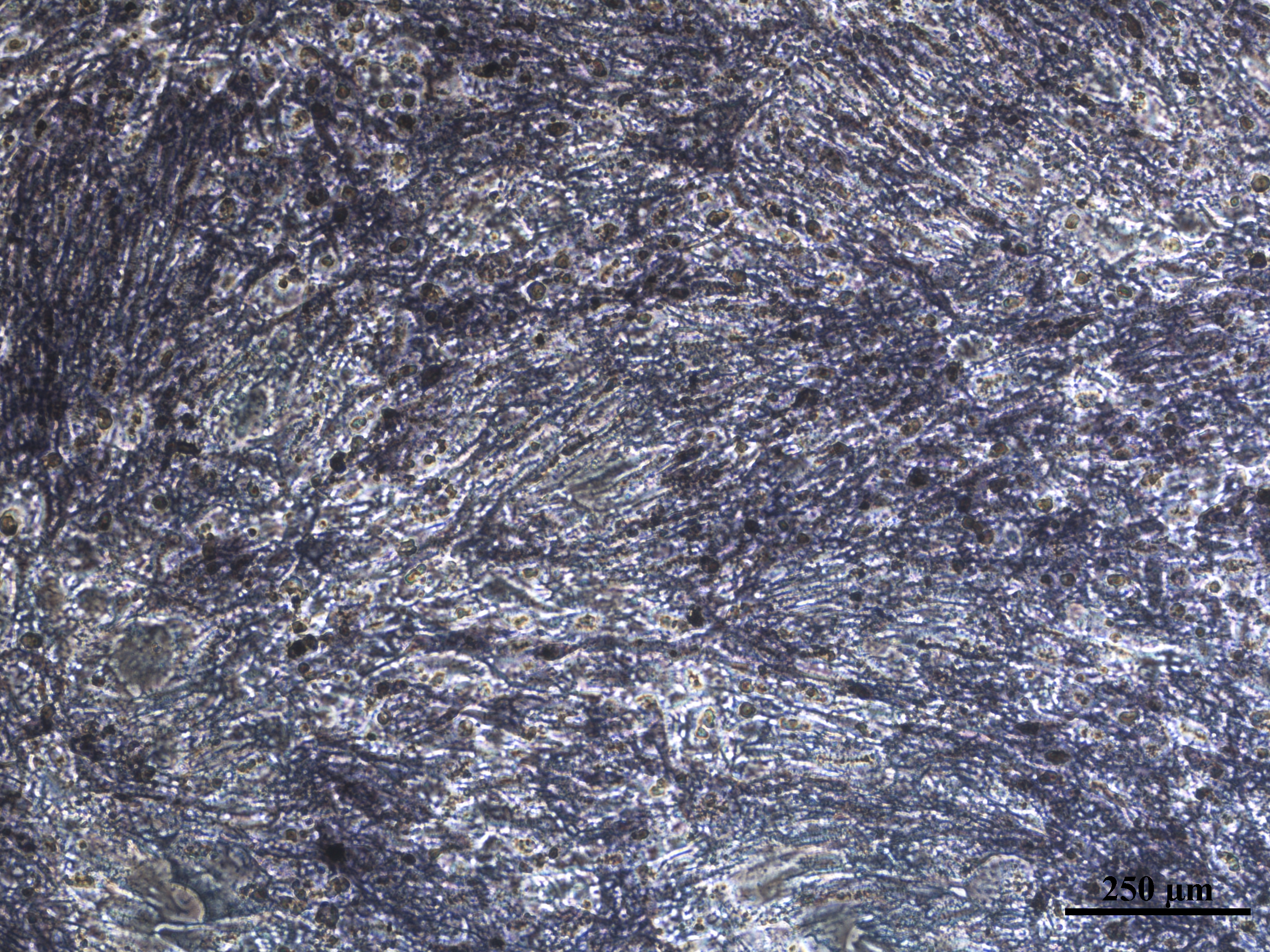

Supplement: Figure 3—source data 1. [file elife-59079-fig3-data1.zip › ALP-GAS5-OE.tif]

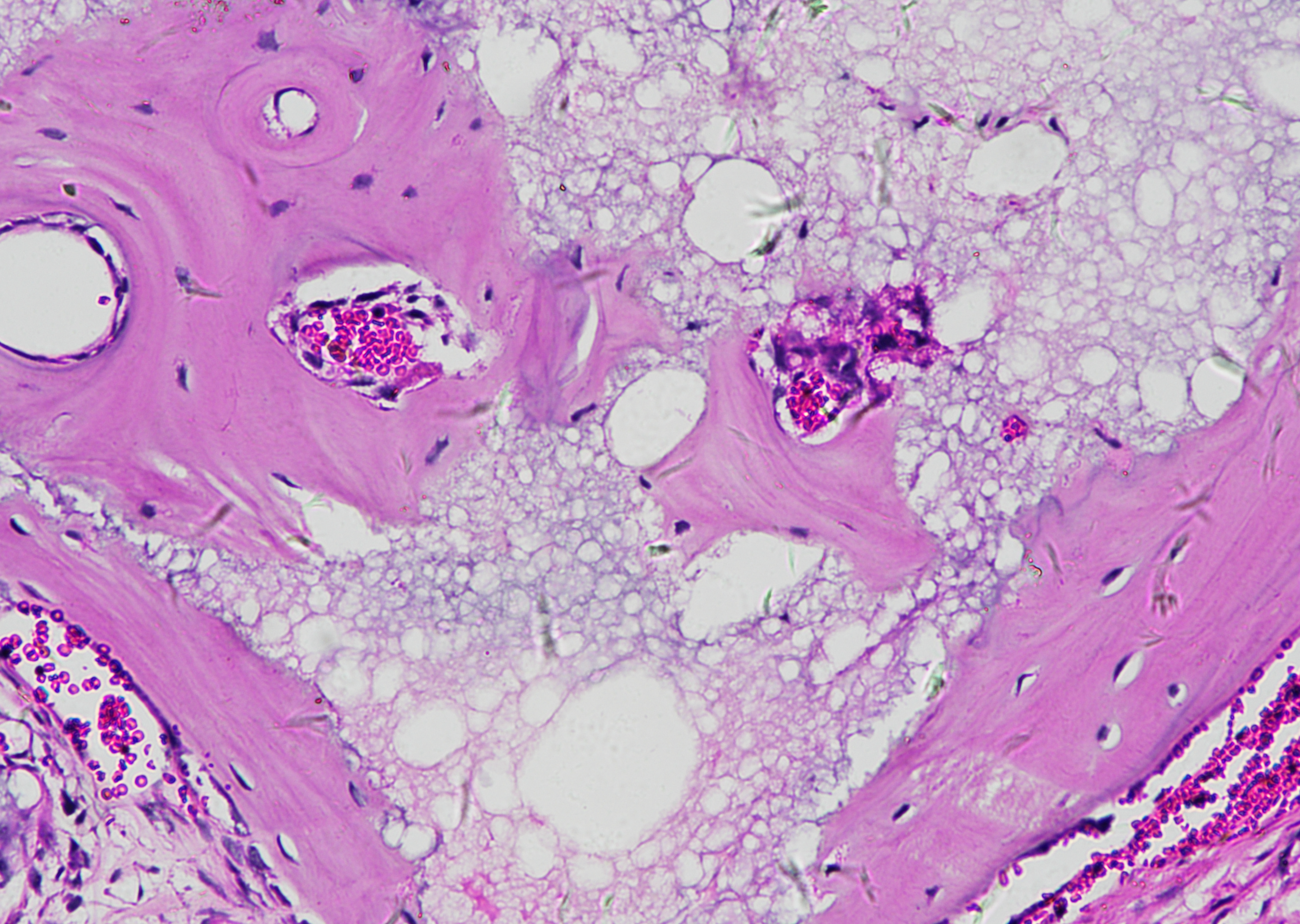

Supplement: Figure 3—source data 1. [file elife-59079-fig3-data1.zip › HE-GAS5-Vector.tif]

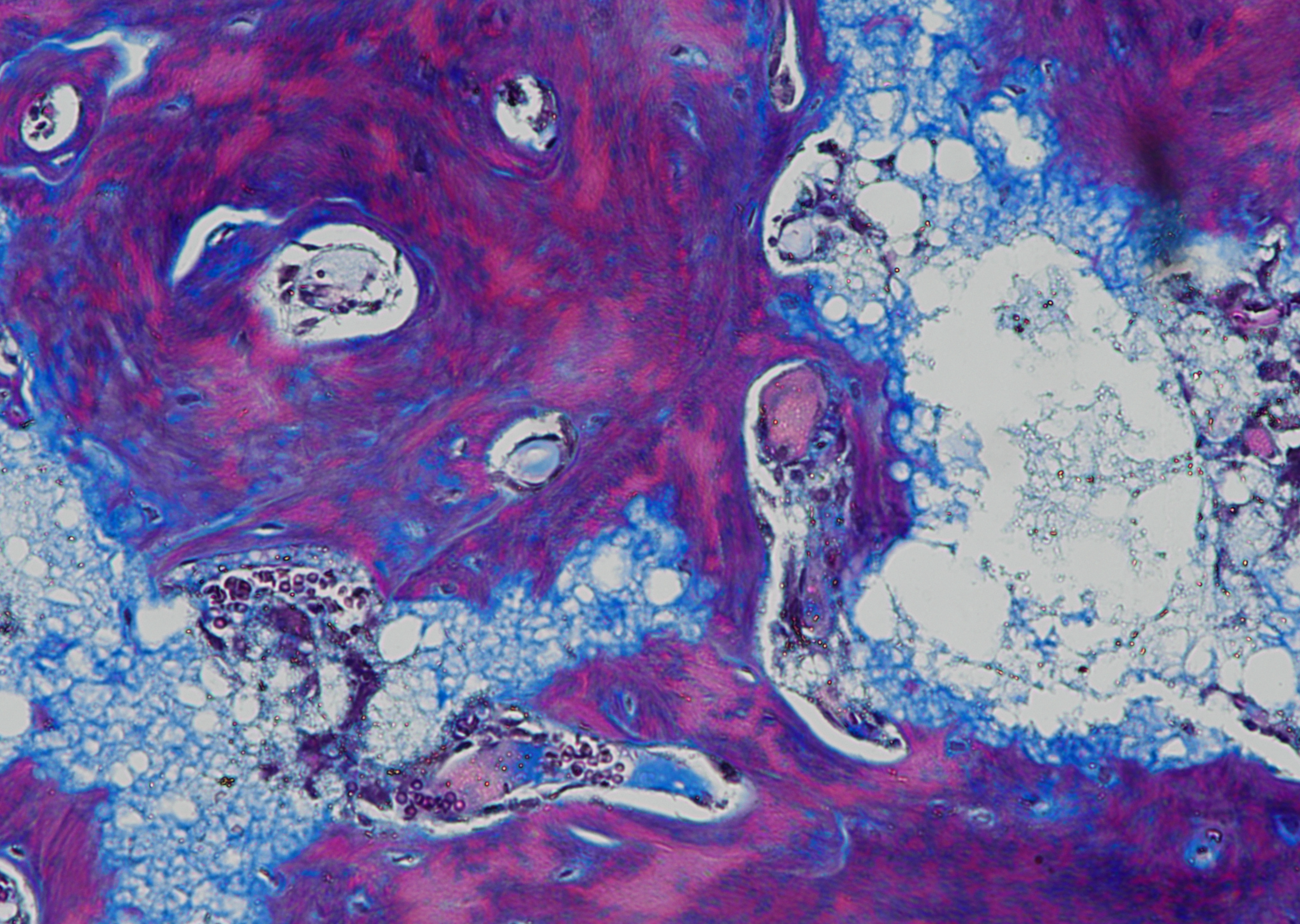

Supplement: Figure 3—source data 1. [file elife-59079-fig3-data1.zip › Masson-GAS5-OE.tif]

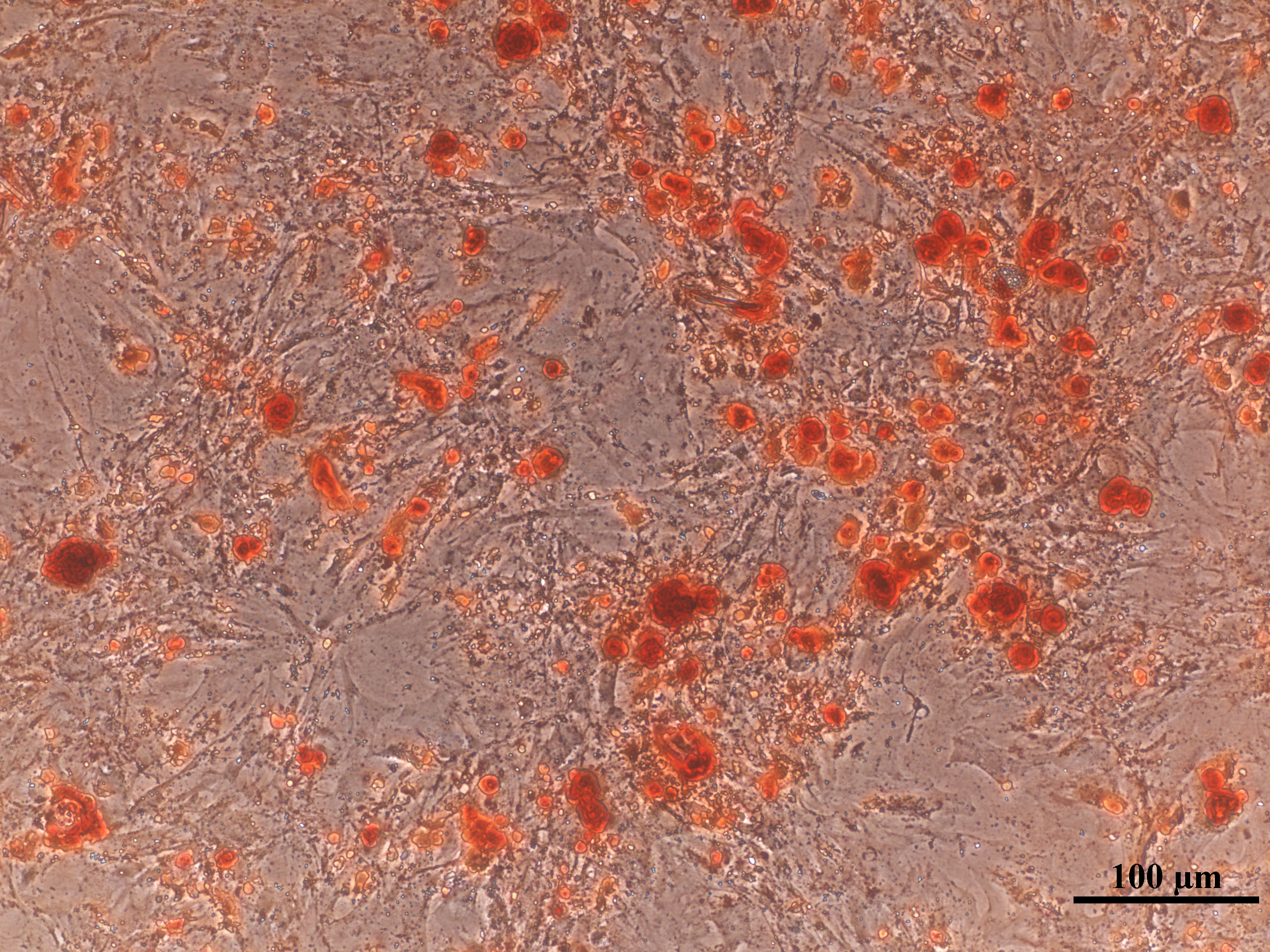

Supplement: Figure 3—source data 1. [file elife-59079-fig3-data1.zip › ARS-GAS5-Vector.tif]

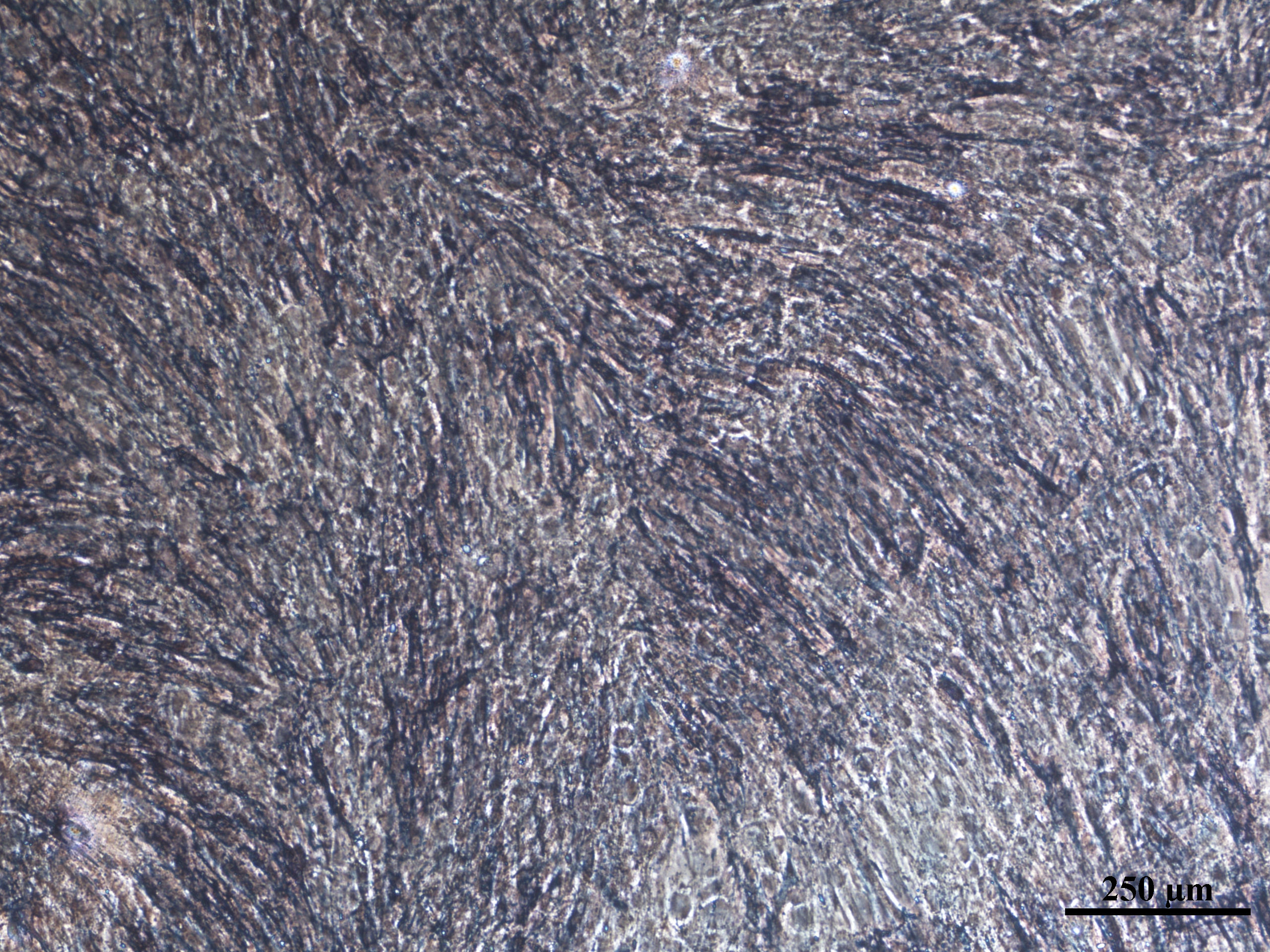

Supplement: Figure 3—source data 1. [file elife-59079-fig3-data1.zip › ALP-GAS5-Vector.tif]

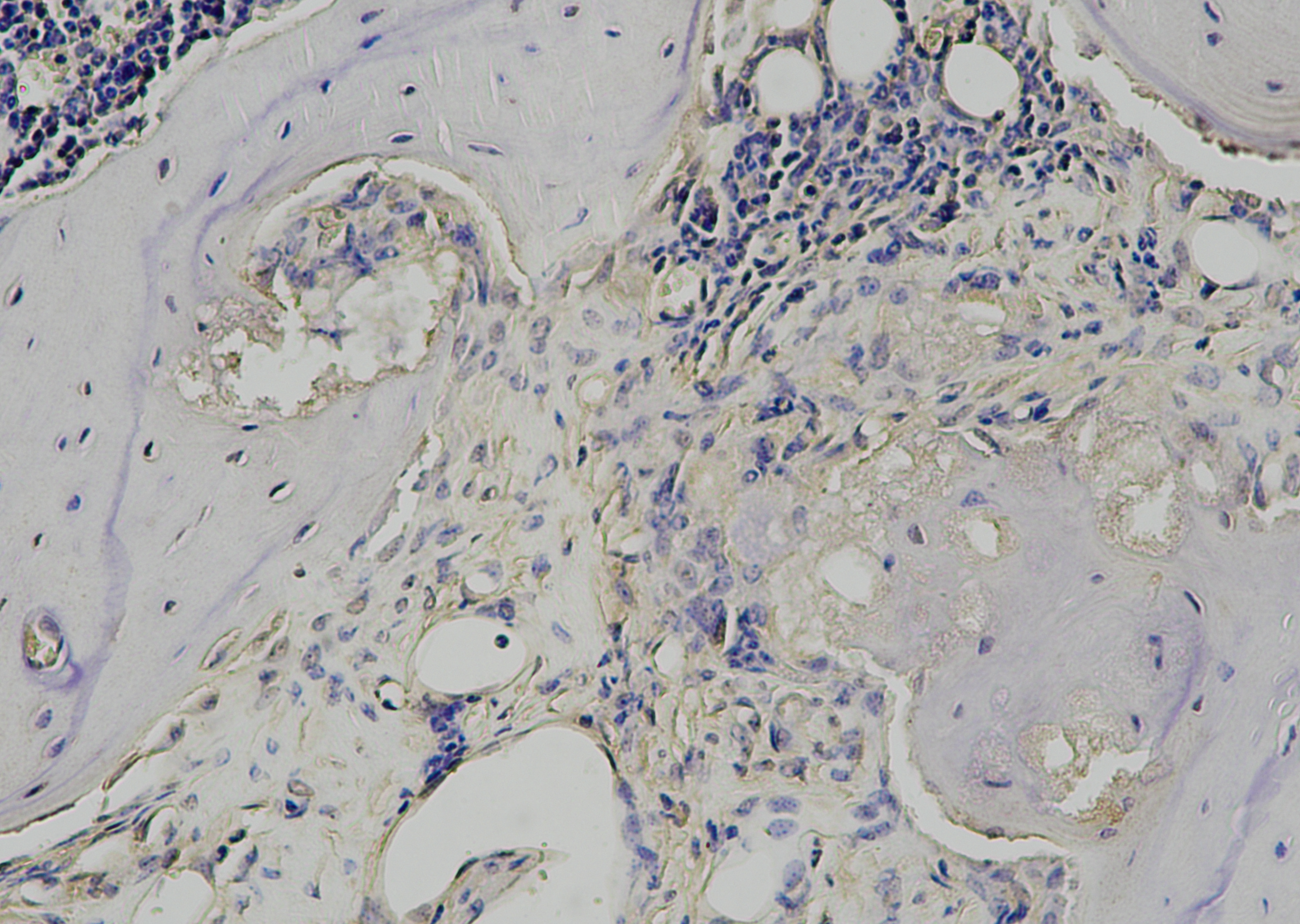

Supplement: Figure 3—source data 1. [file elife-59079-fig3-data1.zip › OCN-GAS5-Vector.tif]

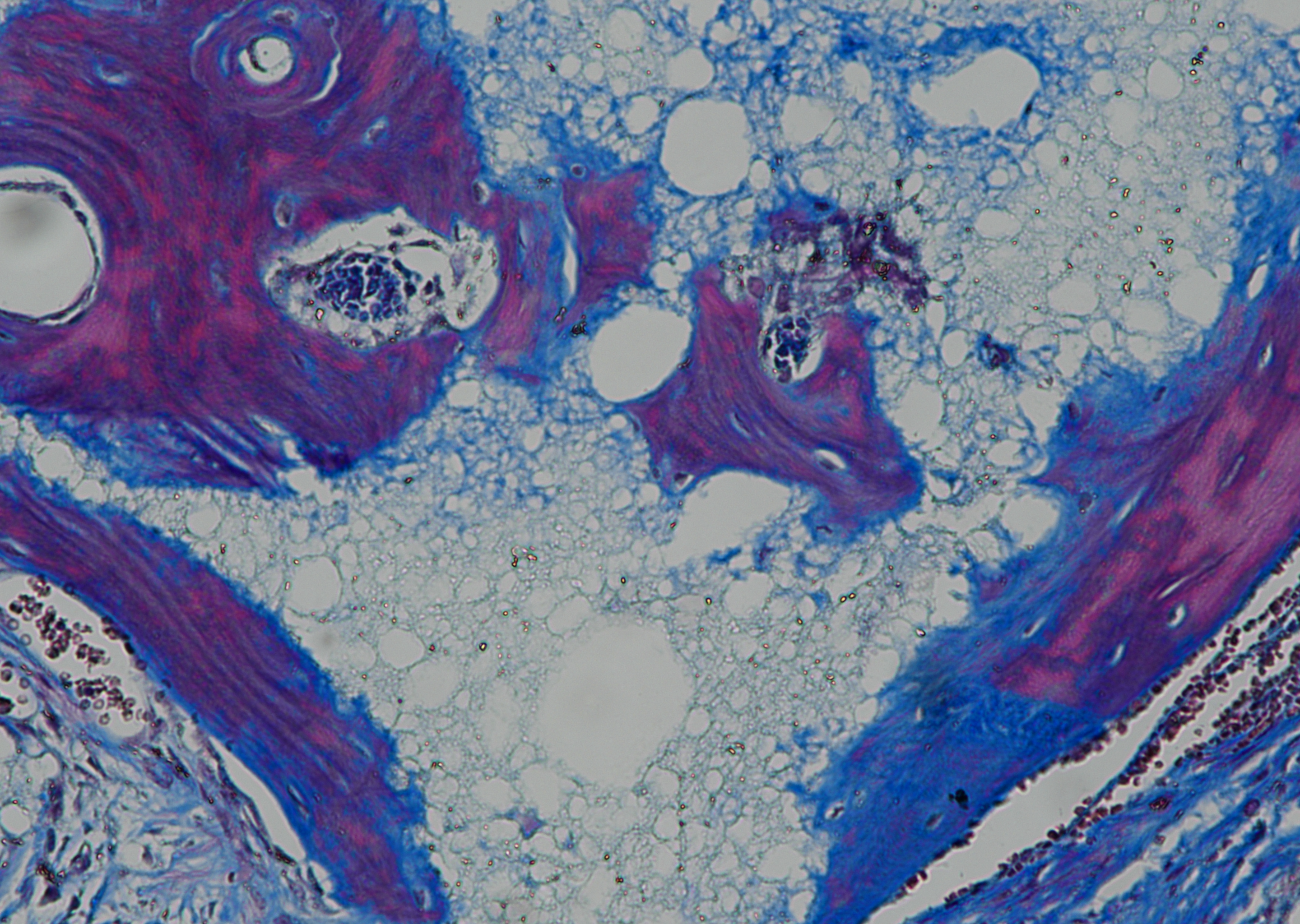

Supplement: Figure 3—source data 1. [file elife-59079-fig3-data1.zip › Masson-GAS5-Vector.tif]

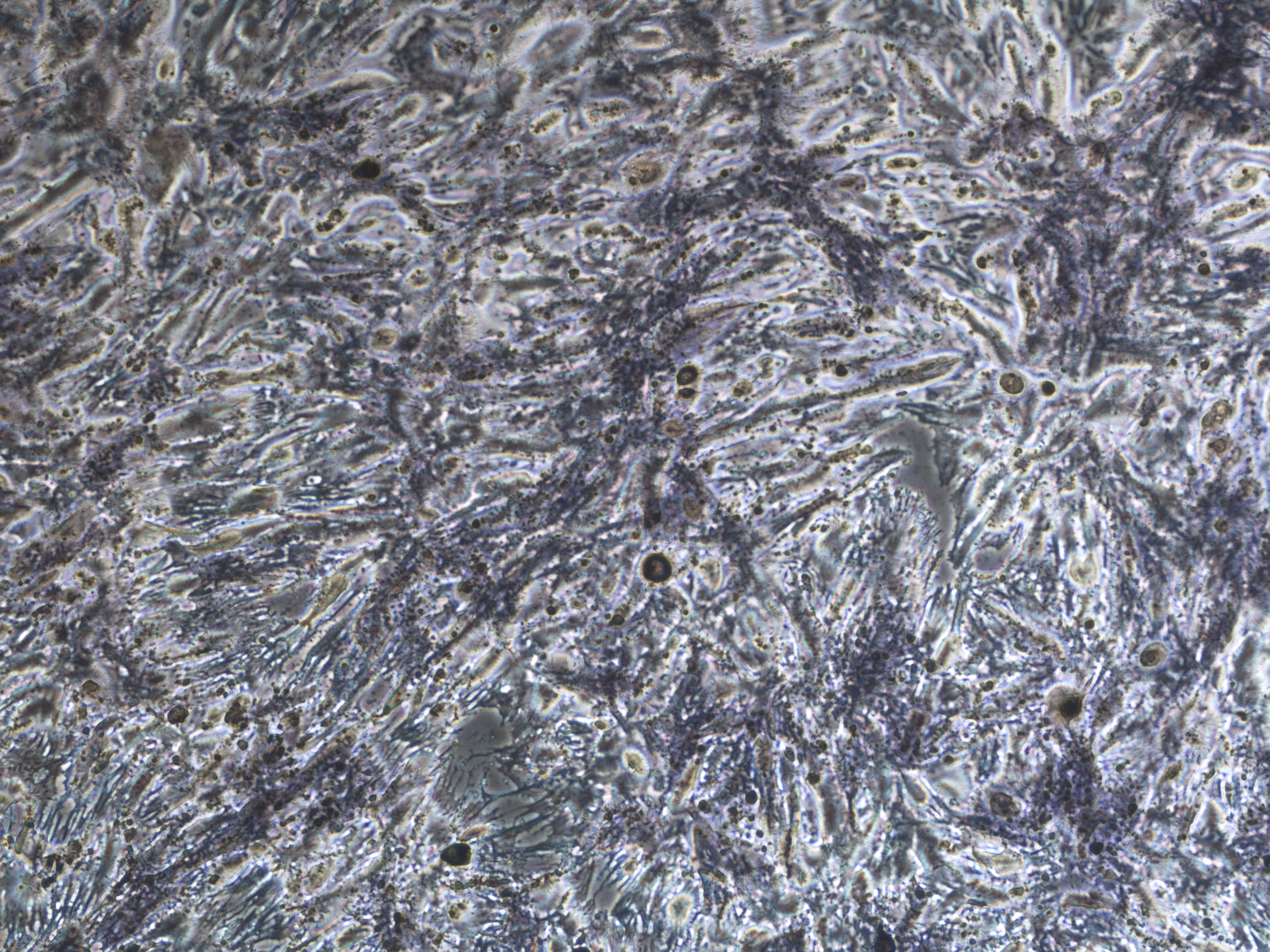

Supplement: Figure 5—source data 1. [file elife-59079-fig5-data1.zip › ALP-NC.tif]

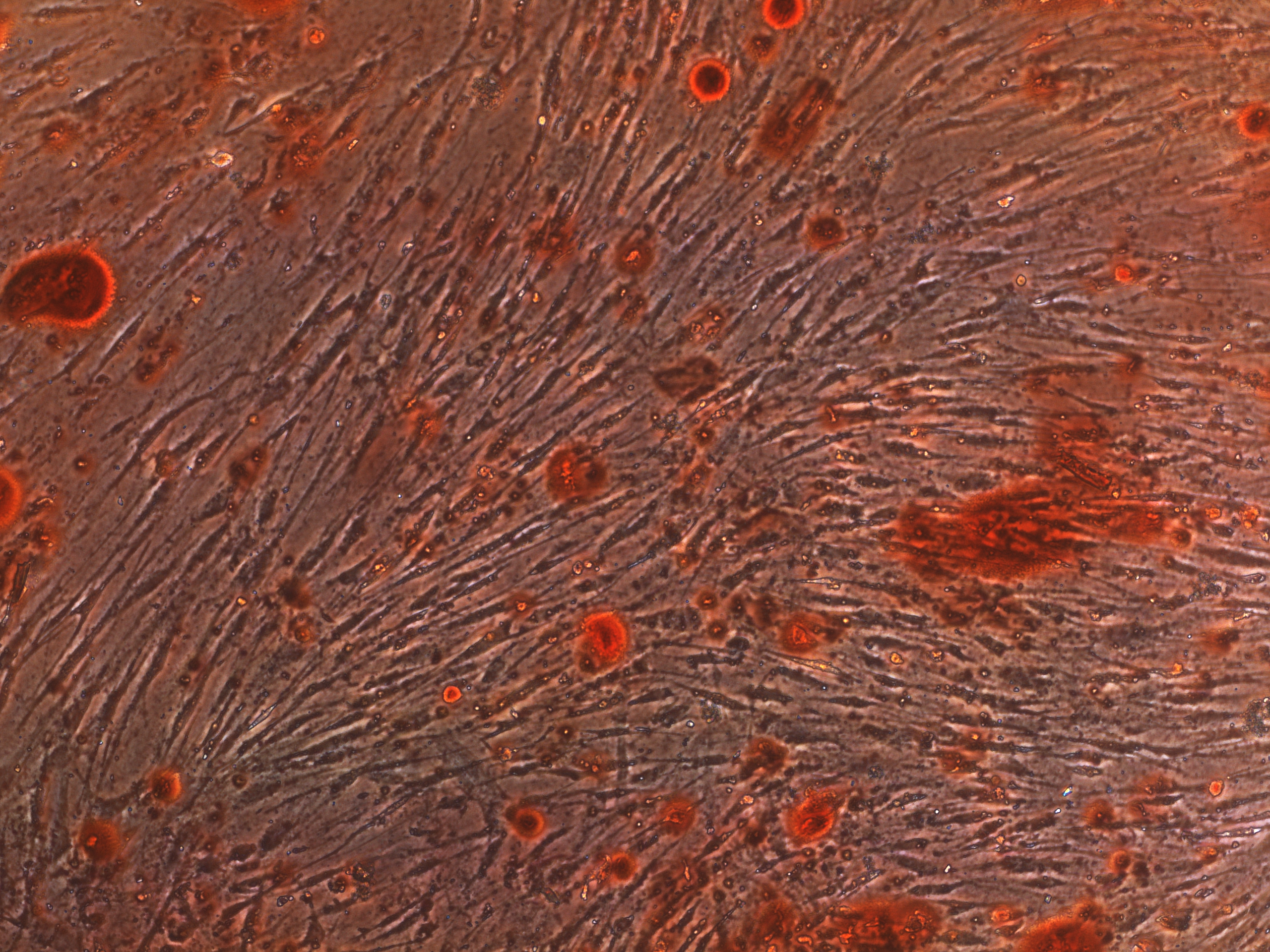

Supplement: Figure 5—source data 1. [file elife-59079-fig5-data1.zip › ARS-NC.tif]

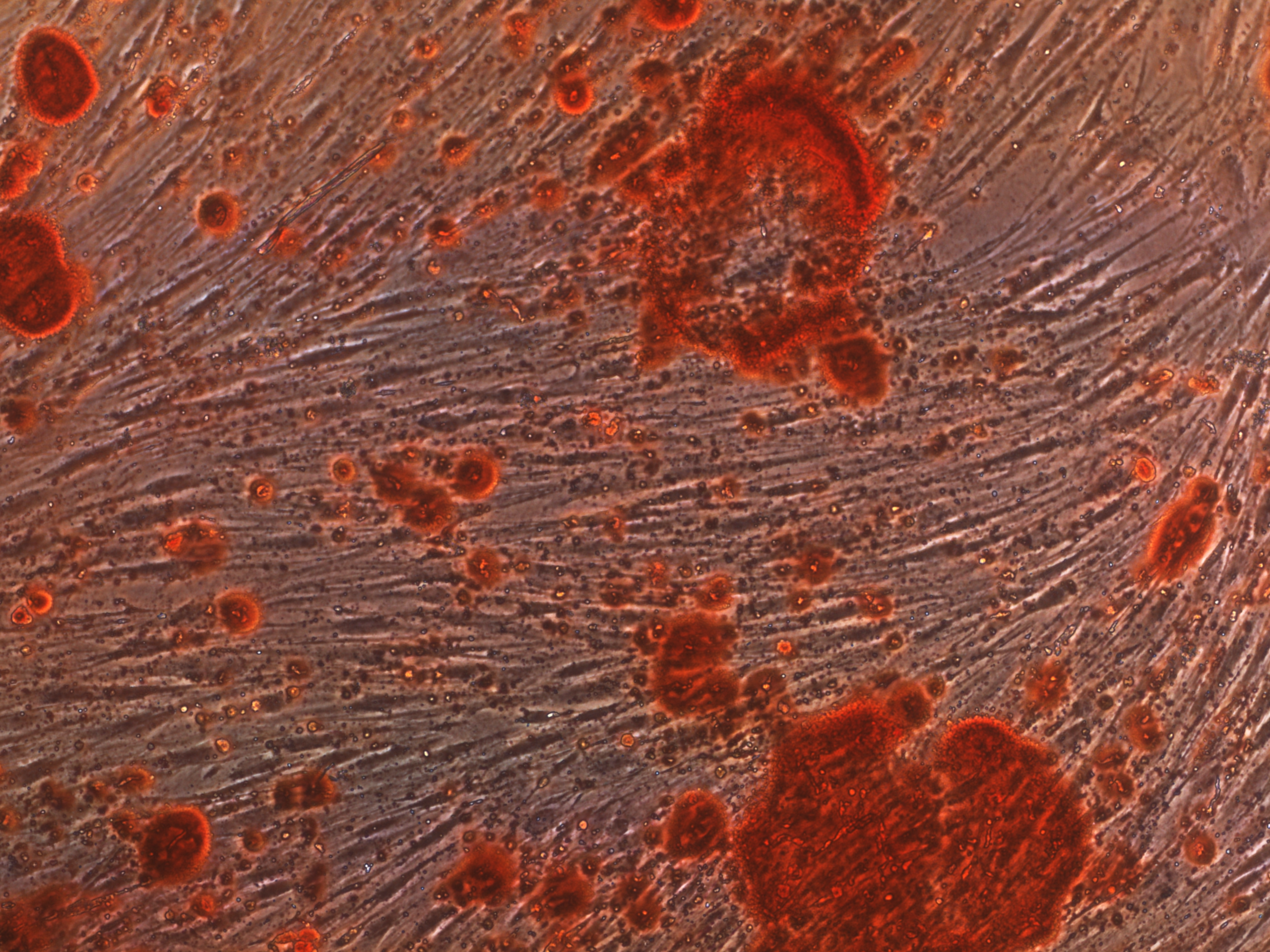

Supplement: Figure 5—source data 1. [file elife-59079-fig5-data1.zip › ARS-GAS5 OE+NC.tif]

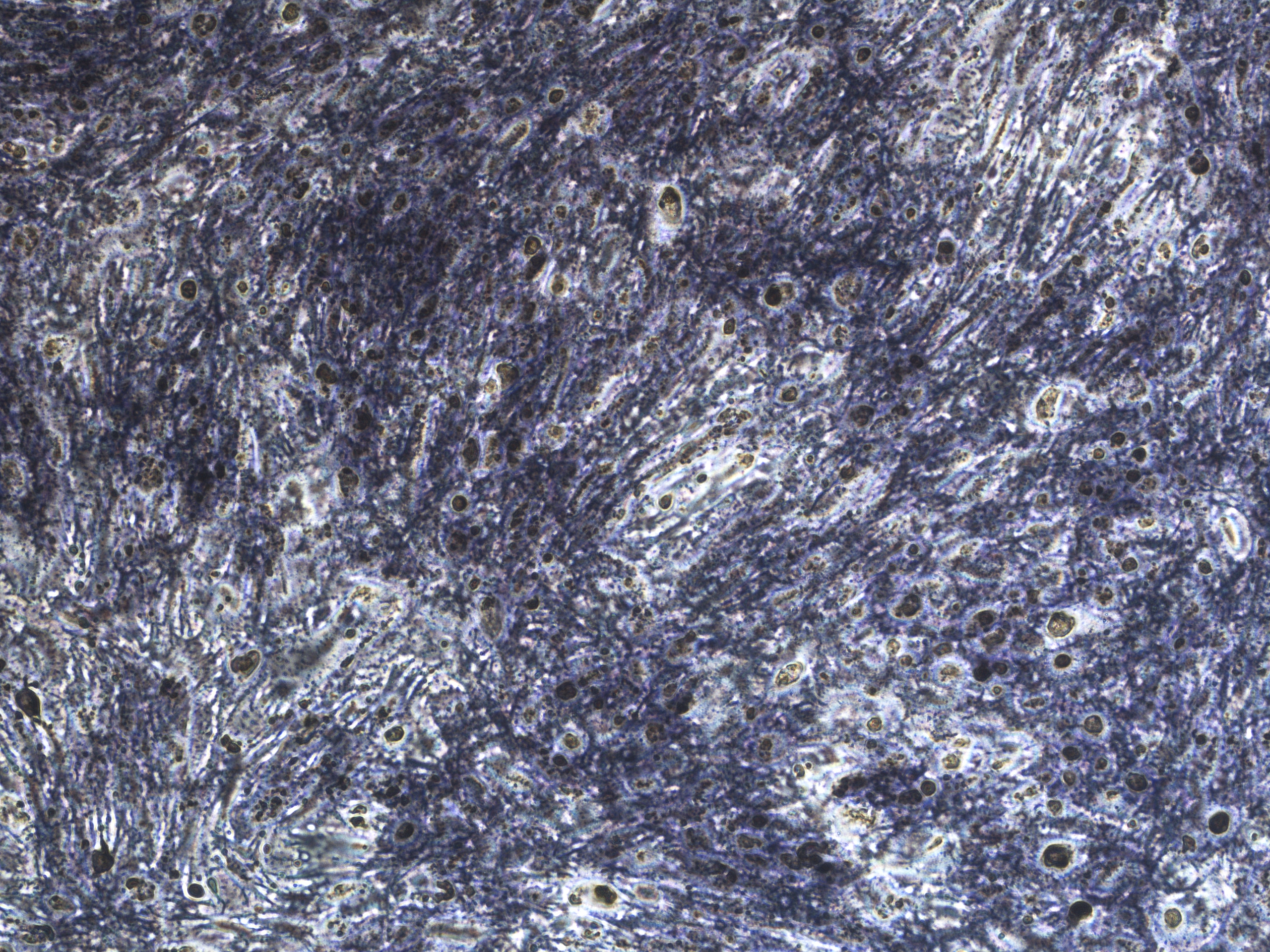

Supplement: Figure 5—source data 1. [file elife-59079-fig5-data1.zip › ALP-GAS5 OE+NC.tif]

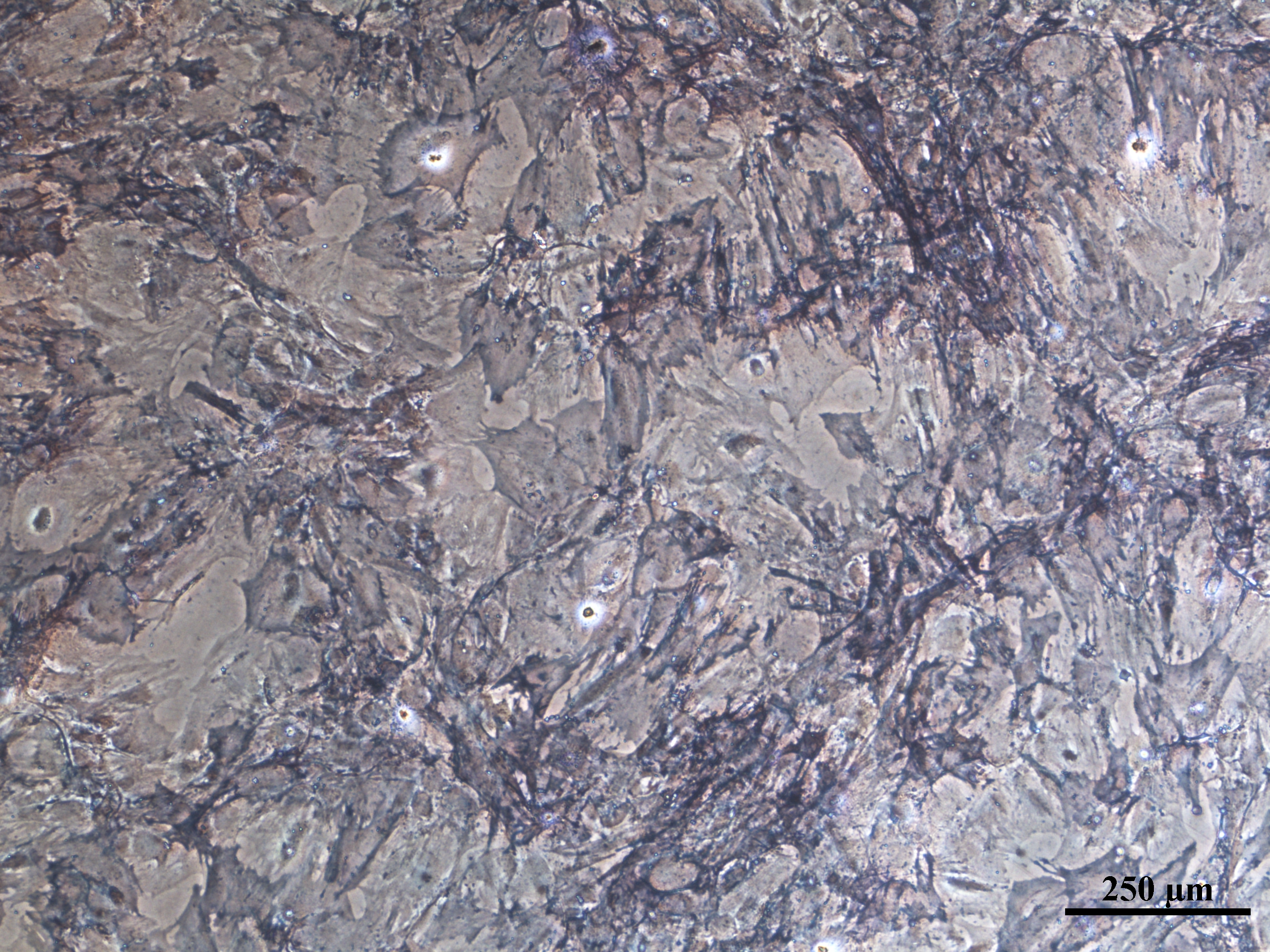

Supplement: Figure 5—source data 1. [file elife-59079-fig5-data1.zip › ALP-GAS5 OE+UPF1 si.tif]

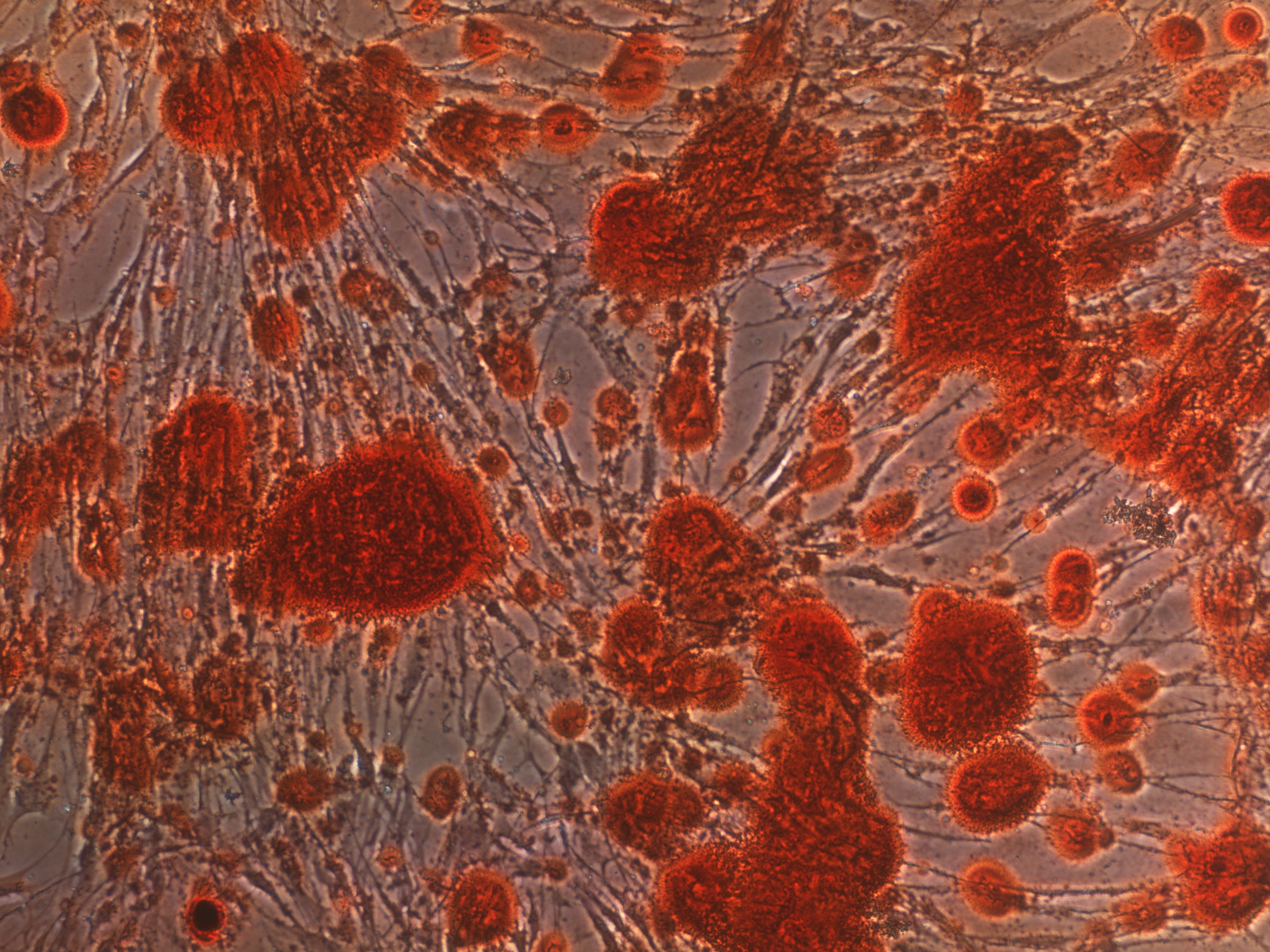

Supplement: Figure 5—source data 1. [file elife-59079-fig5-data1.zip › ARS-GAS5 OE+UPF1 si.tif]
