# Supplementary material for: GAS5 protects against osteoporosis by targeting UPF1/SMAD7 axis in osteoblast differentiation
Source: eLife. 2020 Oct 2;9:e59079. doi: 10.7554/eLife.59079 (PMC7609060; doi:10.7554/eLife.59079)
Supplement: Supplementary file 1. [file elife-59079-supp1.docx]

**Supplementary Table 1:** **Primers of the analyzed genes.**

| Gene Name | Forward Primer | Reverse Primer |
| --- | --- | --- |
| GAPDH | AAGGTGAAGGTCGGAGTCAA | AATGAAGGGGTCATTGATGG |
| GAS5 | AGGTATGGAGAGTCGGCTTG | GCATGCTTGCTTGTTGTGGT |
| ALP | ATGGGATGGGTGTCTCCACA | CCACGAAGGGGAACTTGTC |
| Runx2 | CCGCCTCAGTGATTTAGGGC | GGGTCTGTAATCTGACTCTGTCC |
| OCN | CACTCCTCGCCCTATTGGC | CCCTCCTGCTTGGACACAAAG |
| Col1a1 | GAGGGCCAAGACGAAGACATC | CAGATCACGTCATCGCACAAC |
| SP7 | CCTCTGCGGGACTCAACAAC | AGCCCATTAGTGCTTGTAAAGG |
| UPF1 | CTGCAACGGACGTGGAAATAC | ACAGCCGCAGTTGTAGCAC |
| Smad7 | GGACGCTGTTGGTACACAAG | GCTGCATAAACTCGTGGTCATTG |
| ZBTB37 | GAGCCATCTAAACCAGTTGCG | CATGTGATCCCGGAAATAGGG |
| SERPINC1 | TGAATCCCATGTGCATTTACCG | TGGTAGCAAAGCGGGAATTGG |
| RC3H1 | TCCACAATGGACGGATTTCCT | AACCCAAACTGATGGGCTTTC |
| CACYBP | CTCCCATTACAACGGGCTATAC | GAACTGCCTTCCACAGAGATG |
| TNN | GGAGATGTTCCGCTTCCCTAT | GTCAGCGTCAACCTGAACCA |
| KIAA0040 | CCTCAGACTAAGTGTCCCAGT | GTGGGGTATGCCAGAGACAG |
| TNR | AAGAATTGCTCGGAGCCCTAC | GCTGTACTCGCTGTCACAGAT |
| DARS2 | ATGTGGAGAGTTGCGTTCGTC | TGTTTTGCCTTCGGTACTGAATC |
| CENPL | CACCAGAGTCAACTCCTAGTGC | TCTGCTTCCTGACCGATTCTAA |
| KLHL20 | GCAGGTGTACCAACATTCGAC | GACAAAATGACTCGATGGGCATA |
| ANKRD45 | TGGGGTCGTTTGGAAACTTTG | CCCTGATCCCTTTTCTGTGTC |
| SLC9C2 | AACAGACCTGATTTACTCTGCG | TGAAGCATACAAGCGTTGTGA |
| TEX50 | TATCTGCTTCTTCGGGGAGAG | AAAGTCGCAGCATAGTTTATCCA |
| PRDX6 | GACTCATGGGGCATTCTCTTC | CAAGCTCCCGATTCCTATCATC |
| TNFSF4 | CCAGGCCAAGATTCGAGAGG | CCGATGTGATACCTGAAGAGCA |
| TNFSF18 | AGTGGCTCCCAATGCAAACTA | TATACAGCCGCACCTCAAAAG |
| FASLG | TGCCTTGGTAGGATTGGGC | GCTGGTAGACTCTCGGAGTTC |
| PIGC | ACTATGTGGACCGGCGATTC | CCCTCATCCATATACCACCAGA |
| DNM3 | AGTTCGCCTTGAGATTGAAGC | CGTGTGGGGAATAGACTCGTAAA |
| SUCO | TCATCACCCTGGTACCGAAA | TTCAGTTTGCCGCTGATCCT |
| *Gas5* | GGAAGCTGGATAACAGAGCGA | GGTATTCCTTGTAATGGGACCAC |
| *Upf1* | GTGGCAGCCCCTAATCCAG | GATCTGCTGTGCCGTGATCT |
| *Smad7* | GCATTCCTCGGAAGTCAAGAG | CCAGGGGCCAGATAATTCGT |
