# Supplementary material for: GAS5 protects against osteoporosis by targeting UPF1/SMAD7 axis in osteoblast differentiation
Source: eLife. 2020 Oct 2;9:e59079. doi: 10.7554/eLife.59079 (PMC7609060; doi:10.7554/eLife.59079)
Supplement: Supplementary file 2. [file elife-59079-supp2.docx]

**Supplementary Table 2:** **The siRNA sequences of the analyzed genes.**

| Gene Name | Sense (5’-3’) | Antisense (5’-3’) |
| --- | --- | --- |
| GAS5 siRNA1 | CUUGCCUGGACCAGCUUAAUU | UUAAGCUGGUCCAGGCAAGUU |
| GAS5 siRNA2 | CCAGCACGUUCAUACGAAUTT | AUUCGUAUGAACGUGCUGGTT |
| GAS5 siRNA3 | GCUUCCCUCUGAGUAAAUUTT | AAUUUACUCAGAGGGAAGCTT |
| UPF1 siRNA1 | CAGUGGUGAUGUGCAAGAATT | UUCUUGCACAUCACCACUGTT |
| UPF1 siRNA2 | CACCUGCUGAACUACUAUATT | UAUAGUAGUUCAGCAGGUGTT |
| UPF1 siRNA3 | GACGAGUUUAAAUCACAAATT | UUUGUGAUUUAAACUCGUCTT |
| Negative control | UUCUCCGAACGUGUCACGUTT | ACGUGACACGUUCGGAGAATT |
