# Supplementary material for: GAS5 protects against osteoporosis by targeting UPF1/SMAD7 axis in osteoblast differentiation
Source: eLife. 2020 Oct 2;9:e59079. doi: 10.7554/eLife.59079 (PMC7609060; doi:10.7554/eLife.59079)
Supplement: Supplementary file 3. [file elife-59079-supp3.docx]

**Supplementary Table 3:** **Characteristics of the study subjects.**

|  | Normal control | Hip dysplasia | Osteoporosis Patients |
| --- | --- | --- | --- |
| Number | 8 | 8 | 8 |
| Age (years) | 38.64±10.32 | 42.75±4.95 | 72±5.29 |
| Sex | Female | Female | Female |
| Hight (cm) | 156.45±8.34 | 155.01±4.68 | 156.23±4.09 |
| Weight (kg) | 53.53±8.81 | 56.06±6.12 | 57.4±5.99 |
| BMI (kg/m^2^) | 22.89±3.29 | 23.01±2.82 | 23.49±1.89 |
| Age of menarche (years) | 13.41±1.18 | 13.38±1.06 | 13.25±1.28 |
| Age of menopause (years) | / | 53±2.51 | 54.13±2.75 |
| Lumbar spine BMD (g/cm^2^) | 1.43±0.17 | 1.31±0.15 | 0.64±0.12 |
| Lumbar spine T score | 0.46±0.21 | 0.4±0.13 | -2.84±0.3 |
| Total hip BMD (g/cm^2^) | 1.21±0.32 | 1.13±0.24 | 0.65±0.19 |
| Total hip T score | 0.42±0.26 | 0.35±0.14 | -1.98±0.27 |

Data are shown as the mean ± SD, n=8 in each group. P values for all variables are the result of independent t tests between the control and osteoporosis groups,

BMI, body mass index; BMD, bone mineral density.
