# Supplementary material for: GAS5 protects against osteoporosis by targeting UPF1/SMAD7 axis in osteoblast differentiation
Source: eLife. 2020 Oct 2;9:e59079. doi: 10.7554/eLife.59079 (PMC7609060; doi:10.7554/eLife.59079)
Supplement: Supplementary file 4. [file elife-59079-supp4.docx]

**Supplementary Table 4:** **Characteristics of the 15 healthy donors from Center for Biotherapy, Sun Yat-sen Memorial Hospital.**

|  | **Healthy donors** |
| --- | --- |
| Number | 15 |
| Age, year | 25.6±4.3 |
| No.(%) male | 8(64%) |
| No.(%) of syphilis infection | 0 |
| No.(%) of gonorrhea | 0 |
| No.(%) of HIV infection | 0 |
| No.(%) of hepatitis virus infection | 0 |
